# The use of mindfulness-based cognitive therapy for improving quality of life for inflammatory bowel disease patients: study protocol for a pilot randomised controlled trial with embedded process evaluation

**DOI:** 10.1186/1745-6215-14-431

**Published:** 2013-12-17

**Authors:** Mariyana Schoultz, Iain M Atherton, Gill Hubbard, Angus JM Watson

**Affiliations:** 1 Centre for Health Science, School of Nursing, Midwifery and Health, University of Stirling, Inverness, Scotland; 2 Raigmore Hospital NHS Highland, Inverness, Scotland

**Keywords:** Mindfulness-based cognitive therapy, Inflammatory bowel disease, Crohn’s disease, Ulcerative colitis, Quality of life, Pilot randomised controlled trial

## Abstract

**Background:**

Inflammatory bowel disease (IBD) is a chronic condition with an unpredictable disease course. Rates of anxiety and depression among IBD patients in relapse (active disease symptoms) as well as in remission are higher than in the general population. Previous studies suggest that the prolonged effect of pain, anxiety, distress and depression have a detrimental effect on patients’quality of life (QoL). Poor QoL in itself is associated with further symptom relapse. Mindfulness based cognitive therapy (MBCT) is a psychological group intervention that has the potential to improve QoL. When used in other chronic conditions, it demonstrated reduced negative effect from pain and psychological factors at completion of an 8-week MBCT course. The effect of MBCT has never been researched in IBD. The aim of this study is to obtain the information required to design a full scale randomised controlled trial (RCT) that will examine the effectiveness of MBCT in improving quality of life for IBD patients.

**Methods/Design:**

This is an exploratory RCT with embedded process evaluation. Forty IBD patients will be recruited from NHS outpatient gastroenterology clinics and will be randomised to either a MBCT (intervention) group or to a wait-list (control) group. All participants will undergo 16 h of structured group training over an 8-week period, with the control group starting 6 months later than the intervention group. Primary outcomes are recruitment, completion/retention rates and adherence and adaptation to the MBCT manual for IBD patients. The secondary outcome is to assess the feasibility of collecting reliable and valid data on proposed outcome measures such as quality of life, anxiety, depression, disease activity and mindful awareness. The process evaluation will use a survey and focus groups to assess the acceptability of the intervention and trial procedures for IBD patients.

**Discussion:**

The outcomes of this study will help define the barriers, uptake and perceived benefits of MBCT program for IBD patients. This information will enable the design of a full-scale study assessing the effect of MBCT on quality of life for IBD patients.

**Trial registration:**

Current Controlled Trials: ISRCTN27934462

## Background

Inflammatory bowel disease (IBD) is a group of relapsing chronic gastrointestinal conditions characterised by inflammation of the gut [1,2]. The condition affects the normal function of the gastrointestinal system, resulting in symptoms such as bloody diarrhoea, vomiting, severe pain and malnutrition [3,4]. It affects approximately 250,000 people in the UK and 28 million worldwide with its incidence rising [5,6]. The two main types are Crohn’s disease (CD) and ulcerative colitis (UC). While there is a concern about the increased risk of colorectal cancer in IBD patients [7], the main burden for patients is the relapsing course of the disease and its detrimental impact on psychosocial functioning and quality of life [8].

### Anxiety, depression and quality of life in IBD

Anxiety and depression rates among IBD patients are higher than in the general population even in remission (when symptoms are settled) [9,10]. Previous studies have suggested that the prolonged effect of pain, anxiety, distress and depression have detrimental effects on quality of life (QoL) [11]. Poor QoL is further associated with symptoms relapse and additional clinical difficulties such as tissue inflammation [12,13]. Anxiety, depression and relapse thus links together in a self-perpetuating cycle with pernicious implications for those affected.

### Use of medication for management of anxiety, depression and pain and its limitations

Evidence suggests that the use of antidepressants can have a positive effect on QoL and symptom management for IBD patients by reducing anxiety and depression [14-16]. This is encouraging. However, some patients using antidepressants have reported unpleasant side effects while others have reported that the antidepressants have no effect on their low mood or anxiety [17-19].

The use of painkillers for pain management also has its limitations. There are subgroups of IBD patients who irrespective of using anti-inflammatory and pain medication continue to suffer severe discomfort, again with a knock-on effect and making symptom exacerbation more likely [20].

In addition, poor medication compliance is frequently reported among IBD patients, with up to 40% of patients regularly omitting their prescribed medications [21,22]. Pharmacology is thus very limited. The search for alternative evidence-based approaches is a pressing concern.

### Mindfulness based cognitive therapy

Mindfulness based cognitive therapy (MBCT) is a non-pharmacological psychological group program designed by Segal et al. [23]. The MBCT program is largely based on the mindfulness-based stress reduction (MBSR) program developed by Jon Kabat- Zinn for coping with stress and chronic illness [24]. Both programs involve teaching individuals various stress management, relaxation, self-care and self-help techniques in a systematic way over an 8-week period. In both, the core skill taught is mindfulness, which uses meditation practice to increase attention and awareness [25]. The working definition of mindfulness in the program is: ‘The awareness that emerges through paying attention on purpose, in the present moment, and nonjudgmentally to the unfolding of experience moment by moment’ [26]. The main difference to the MBSR program is that MBCT has combined cognitive therapy exercises with the mindfulness skill. This combination is believed to further enable patients to increase their awareness and facilitate early recognition of any recurring unhelpful thinking patterns often associated with depressive symptoms and anxiety [23]. Hence, the National Institute for Health and Care Excellence guidelines makes a recommendation for the use of MBCT program as a psychological intervention in the ‘clinical management of persistent sub threshold depressive symptoms or mild, moderate or severe depression in adults (including people with a chronic physical health problem)’ [27].

Although the exact mechanism of how mindfulness-based interventions work is not yet fully understood, the evidence so far suggests that at program completion, participants would experience reduced negative effects from pain, distress, anxiety and depressive symptoms. For example, a systematic review and a meta-analysis of the effectiveness of mindfulness-based interventions on anxiety, depression and psychological distress in patients with chronic pain conditions [28] and chronic medical conditions [29] such as fibromyalgia, cardiac and cancer patients, have shown positive effect on anxiety, depression and psychological distress.

An observational study examining the relationship between mindfulness, QoL, depression and anxiety in patients with ulcerative colitis found a positive association between increasing mindfulness and reducing depression and anxiety [30]. However, MBCT and its effect on QoL has never been researched in a RCT with both Crohn’s and ulcerative colitis patients.

Thus, the MBCT program may be the therapy that can provide a relief from the negative effects of a lifelong management of the disease for IBD patients.

### Hypothesis

The hypothesis is that MBCT will improve QoL scores for IBD patients as well as improve anxiety and depressive symptom scores. This hypothesis is based on previous studies using mindfulness based programs in other chronic condition populations. Hence, the proposed pilot study is the first step towards testing the hypothesis in a definitive RCT and gathering the necessary knowledge to close the existing evidence gap regarding the usefulness of MBCT for IBD patients.

### Aims and objectives

The overall aim of this study is to pilot the MBCT program with IBD patients in a RCT and examine the feasibility of running a large RCT testing the above hypothesis.

The specific objectives of the pilot are:

1. To determine the feasibility of conducting a large-scale RCT of group MBCT for improvement of IBD QoL.

2. To adapt the intervention manual devised by Segal et al. [31], outlining how to carry out an MBCT program for IBD patients.

3. To use data arising from differences between MBCT and control arm to inform a power calculation for sample size of a definitive RCT.

4. Estimate trial eligibility, recruitment (percentage of IBD patients who consent to the trial) and completion/retention rates (percentage of participants completing the trial).

5. To embed a process evaluation within the pilot trial to assess the acceptability of the intervention and trial procedures for IBD patients, such as:

a. Acceptability of recruitment, randomisation and consent procedure

b. Acceptability and feasibility of collecting reliable and valid data on primary and secondary outcomes (including follow-up at 6 months)

c. Fidelity of intervention.

d. Acceptability of length of intervention

e. Appropriateness/suitability of the intervention used

f. Barriers to attendance

g. Expectations about intervention

h. Perceived impact on QoL

## Methods and design

### Design

This exploratory trial is designed in two phases. Phase 1 is a two-arm pilot RCT (MBCT treatment *vs*. wait-list control group) with three assessments (baseline, post treatment and 6 months). Phase 1 will assess eligibility, uptake, drop-out rates and sample size calculation as well as adaptation and adherence to MBCT manual. This design is consistent with similar studies where a non-pharmacological intervention is investigated in IBD population [32-38].

Phase 2 is a process evaluation assessing the feasibility and acceptability of the intervention, primary and secondary outcomes and trial procedures as well as barriers to attendance and perceived benefits to patients. This design is in line with the Medical Research Council (MRC) framework for the development and evaluation of complex interventions to ensure that both the intervention and trial procedures are optimised and can be incorporated into routine clinical practice [39].

### Setting and recruitment

The study will take place across two health board areas in the north of Scotland, an area comprising of approximately 800,000 people living across a large geographical area including urban and remote rural locations. Recruitment will focus on gastroenterology outpatient clinics.

### Phase 1 - Pilot RCT

#### Participant selection

Less than one-third of RCTs recruit to proposed number [40]. Assessing recruitment is a key component of this study. Ensuring an effective recruitment strategy is thus important. Accordingly, the recruitment strategy devised in this study draws on best evidence [41] tailored to the specific requirements of people with IBD. Specific aspects of the recruitment strategy were developed in collaboration with all parties involved in the care of IBD patients, namely specialist IBD nurse, gastroenterology clinicians and research nurses.

Clinical staff at participating gastroenterology outpatient clinics will identify people who meet the study’s inclusion criteria. They will approach consecutive patients in clinics or by sending an invitation letter with study information and research team contact details. Interested participants will then contact the researcher by telephone or email to register interest. A screening visit with the researcher will then be arranged. Informed written consent will be obtained at the first visit by a member of the research team.

#### Inclusion criteria

1. Be able to verbally communicate and write in English (English does not have to be their first language).

2. Able to give informed consent.

3. Age of 18 years or over (no upper limit).

4. Confirmed diagnosis of Crohn’s disease or ulcerative colitis (by clinician).

5. Ability to do light exercise (for example, to lift arms above the head or bend knees) because part of the practices in the program require this movement.

6. Able to commit to attend the eight sessions (participants should consider their personal circumstances to assess if this is practical and feasible for them).

7. To be able to commit to do home practice of up to 45 minutes daily over the 8 weeks of the study (this is a core component of the program).

8. No change of antidepressants (dose or type) within the last 3 months. Any change of antidepressants within the last 3 months might interfere with the program.

9. Participants will have to be in remission of symptoms.

#### Exclusion criteria

Anyone not meeting the above criteria by definition will be excluded from the study. In addition, the following exclusion criteria will apply:

1. Major psychiatric illness. The treatment for a major psychiatric illness may interfere with the program.

2. Active alcohol or drug dependency. Any alcohol or drug dependency may interfere with the program.

3. Scheduled for major surgery in the next 3 months. Any scheduled surgery within the next 3 months will interfere with the program schedule.

4. Participation in a pharmacological study or psychological intervention study within the last 6 months or intention to participate in a pharmacological study during the duration of this study. Both will interfere with the program.

5. Have recently (within the last 3 months) been prescribed antidepressants. Any change of antidepressant in the last 3 months may interfere with the program.

6. With exacerbated symptoms (acute phase). Having exacerbated symptoms will make it very difficult for participants to attend the two hourly sessions or to commit to the home practice. This could cause extra unwanted stress for the patient.

#### Randomisation

Randomisation will be performed using a dedicated software solution after participants have given written consent and baseline data have been collected. Group allocation will be to ‘MBCT group’ or ‘wait-list control group’ in a 1:1 ratio. To ensure similarity between the groups, randomisation will take account of gender and type of disease. The randomisation will be carried out by an independent statistician.

#### Minimising bias

Participants will self-complete all the questionnaires. Data entry will be done by the lead researcher and independently checked by a second person. Data analysis will be done by two researchers independently and differences will be rectified by a third person.

#### MBCT intervention

The MBCT program used in this study will follow the manual developed by Segal et al. [31]. In brief, the manual proposes the following format for the eight group sessions:

•Welcome and introduction to the session theme

•A short opening meditation

•A discussion of at-home practice

•An introduction and practice of new exercises

•A group reflection/discussion

•A review of the next weeks’ at-home practice

•A closing sitting meditation

A sample list of activities for session 1 is presented below. The manual also suggests for an additional full day of mindful practice to take place between weeks 6 and 7 (usually on a weekend). In the full day of practice participants will go through all the learned meditations one after another in silence, with the group reflection and discussion taking place at the end of the practice day. An example schedule for a day of mindful practice is presented below. Due to resource constraint, in this study, the full day of practice will be offered to participants after they have completed the 8-week course.

### A sample list of activities for session 1

Theme: Awareness and automatic pilot

1. Establish the orientation of the class

2. Set ground rules regarding confidentiality and privacy

3. Ask participants to pair up and introduce themselves to each other than to the group as a whole, giving their first names and if they wish, saying what they hope to get out of the program

4. The raisin exercise

5. Feedback and discussion of the raisin exercise

6. Body scan practice-starting with short breath focus

7. Feedback and discussion of body scan

8. Home practice assignment:

• Body scan for 6 out of 7 days

• Mindfulness of a routine activity

• Distribute audio files: cd’s for those that not have email and session 1 participant hand-outs.

9. Discuss in pairs:

• Timing for home practice

• What obstacles may arise

• How to deal with them

10.  End the class with a short 2–3 minute focus on the breath.

### Example schedule for a day of mindful practice [35]

9.45-10.00 Arrival

10.00-10.05 Sit in silence

10.05-10.20 Welcome, introduction, ground rules

10.20-10.50 Siting meditation: Breath, body, sounds, thoughts and choiceless awareness

10.50-11.30 Mindful stretching

11.30-12.00 Body scan

12.00-12.05 Instruction for lunchtime: bringing focus on awareness of eating, tasting, chewing, swallowing, slowing down

12.05-13.05 Lunch followed by mindful walk

13.05-13.20 Sitting meditation

13.20-13.50 Walking meditation

13.50-14.20 Mountain meditation

14.20-14.40 Mindful stretching

14.40-15.00 Silent sit or extended breathing space

15.00-15.30 Feeding back experience of day in pairs

15.50-16.30 Large group discussion and close

The program will be delivered by two experienced MBCT practitioners that have been briefed on the key concerns and difficulties that IBD patients experience as well as the nature of the disease. An experienced practitioner will have completed an 8-week MBCT course and maintained a personal practice for at least 1 year. In addition they will have facilitated at least one 8-week MBCT program.

Participants will be encouraged to do daily home practice for the duration of the program and keep a home practice diary. Participants will be provided with guided practice CDs and hand-outs with a written summary for each session and instruction for home practice. Each weekly session will be approximately 120 minutes and will be audio recorded.

#### Wait-list control

The control group will continue to receive their standard care and in addition will receive a leaflet entitled ‘Staying well with IBD’. The leaflet is readily available to download from the Crohn’s and Colitis UK website, but participants in the study will receive a printed copy [42]. After 6 months follow-up data are collected; the wait-list group will have an opportunity to attend a MBCT program if they wish.

#### Data collection

**Figure 1:**
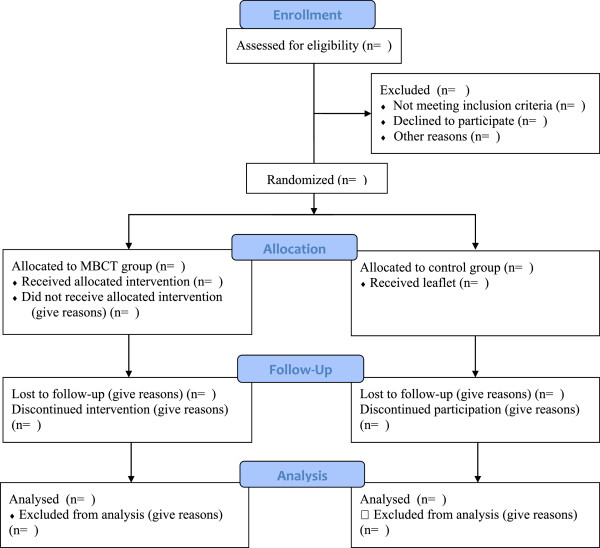
Consort diagram describing flow of patients through study.

A screening and recruitment log will be completed by a researcher to document all patients considered for the study and subsequently included or excluded at each stage of the recruitment process with reasons given. This log will include information such as date when information was given about the study, and date of recruitment and randomisation. The data will inform the estimate for recruitment rates for a full trial and address aims 1 and 4. A full consort diagram of subject flow is presented in Figure 1.

At baseline, demographic information including age, gender, education, income and marital status will be recorded, to allow the success of randomisation to be assessed [43].

The MBCT practitioners will complete an attendance log to document the number of practice sessions attended by each participant. This log will inform the estimated attrition rates for a full trial and will address aim 3.

Semi-structured interviews with the practitioners delivering the MBCT intervention will be conducted to find out if any changes to the manual should be done to accommodate the needs of this patient group. This will address aim 2.

#### Outcomes and assessments

The outcomes for this study are consistent with relevant published studies assessing the use of mindfulness-based program in populations with chronic health problems [44] as well as with studies investigating the effects of non-pharmacological programs on QoL in IBD patients [35,36,45]. They include assessment of disease specific QoL, mood, mindful awareness, disease activity and demographics. All assessment tools used in the trial are validated and reliable. All outcomes will be assessed at baseline, post treatment and 6 months.

#### Proposed primary outcome

##### Quality of life

All participants will be required to complete the disease specific IBD quality of life (IBDQoL) questionnaire consisting of 32 questions. The questionnaire has four domains, including bowel symptoms, systemic symptoms, emotional factors and social factors. Within the questionnaire, participants will rate their symptom experience over the previous 2 weeks. Low scores indicate more severe disease activity and/or higher emotional and social dysfunction. A relatively good correlation between the IBDQoL and a widely used measure of disease activity, the Crohn’s Disease Activity Index is reported (*r* = -0.67; *P* <0.001) [46,47].

#### Proposed secondary outcomes

##### Anxiety

Anxiety will be measured by the State and Trait Anxiety Inventory (STAI) Y1 and Y2 form, consisting of 40 questions on a self-report basis. The inventory measures two types of anxiety: anxiety related to an event and anxiety level as a personal characteristic. Higher scores are positively correlated with higher levels of anxiety [48]. This tool is widely used to measure anxiety and regarded as highly reliable [13].

##### Depression

Low mood and depression symptoms will be measured with the Beck’s depression inventory (BDI-II), consisting of 21 group of statements referring to the last 2 weeks. Each answer is being scored on a scale value of 0 to 3. Higher total scores indicate more severe depressive symptoms. Previous studies indicate the internal consistency of the BDI-II is high [49,50].

##### Disease activity

Disease activity will be measured with the eight-item questionnaire Crohn’s Disease Activity Index (CDAI) for Crohn’s patients [51,52] and six-item questionnaire Simple Clinical Colitis Activity Index (SCCAI) for ulcerative colitis patients [53]. Both tools are widely used to measure disease activity with IBD patients.

##### Mindful attention

Mindful attention will be measured with the 15-item scale Mindful Attention Awareness Scale (MAAS). This scale consists of collection of statements about everyday experiences graded by participants, using a scale of 1 to 6 which indicates how often each experience occurs. Higher scores reflect higher levels of dispositional mindfulness. The validation of this tool has been examined in a series of studies indicating strong psychometric properties and validity [54,55].

### Phase 2 - Process evaluation

To address aim 5 and examine the implementation and receipt of the intervention and trial procedures, a detailed process evaluation will be undertaken. This evaluation process will assess the acceptability of recruitment, randomisation and consent procedure for patients, acceptability and feasibility of collecting reliable and valid data on primary and secondary outcomes, fidelity of protocol, acceptability of length of intervention for patients, appropriateness/suitability of the intervention used, barriers to attendance, expectations about intervention and perceived impact on QoL.

Observations of MBCT training sessions will be undertaken by audio recording of all sessions to assess if specific components of the protocol are delivered and to use the qualitative data to assess the appropriateness of the intervention used. These data will address aim 5c.

All participants will be asked to self-complete a postal survey asking their views on research procedures such as consent and randomisation procedures and reliability of questionnaires. The MBCT group will answer further questions on expectations, acceptability, appropriateness and perceived impact of the intervention as well as the length of the program. These data will address aims 5a, 5e, 5f, 5g and 5h.

A post-intervention focus group will be facilitated to further explore the participants’ views and experiences of the MBCT program and trial procedures, using the themes from the survey. The focus groups will be audio recorded. A list of topic guides for the focus group is presented below.

### List of topic guides for the focus groups

  Expectations

1. Did you have any expectations regarding the self-help program?

2. If yes, what were they?

3. Were your expectations met/ unmet in any way? And How?

Length and difficulty of program

4. Was the length of eight weeks for the program acceptable?

5. Did you find the program difficult to follow?

6. Please tell us which specific parts of the program you found difficult to follow.

Potential benefits

7. Do you think this program has brought any benefit to you?

8. Please tell us how you think the program benefited you.

9. Do you think you will continue to use some of the techniques you learned in the program?

Recruitment and randomisation

10. Do you think the process of recruitment was acceptable?

11. Do you think the patient information sheet was easy to understand?

12. Any suggestions to make it clearer to understand

13. Did you feel comfortable with the process of consent and randomisation?

Questionnaires

14. Was filling the questionnaires at the start and at the end of the eight weeks too much of a burden?

15. Can you please tell us what difficulties you came across with the questionnaires and perhaps any suggestions of how to overcome those difficulties

Barriers to attending

16. Were there any barriers to attending the program?

17. Can you please tell us what you think were barriers to attendance?

What did you enjoy?

18. Did you enjoy the program?

19. Which parts did you most enjoy?

Availability of MBCT program

20. Do you think this program should be made available to IBD patients through NHS?

### Ethical and research and development approval

A favourable ethical opinion was obtained from NRES Committee for North of Scotland on 8th April 2013 (REC ref 13/NF/0018). NHS Highland and NHS Grampian R&D Management Approval was obtained on 9 April 2013 and 14 September 2013, respectively.

### Sample size

The nature of this study is a pilot RCT. Thus, a formal sample size calculation was not performed. The determined sample size of *n* = 40 was calculated based on the estimated number of participants expected to complete the 8-week program.

This number was achieved by the following calculation. Recommendation for pilot sample size calculation is 30 [56,57]. Literature suggest that attrition rate of approximately 25% is to be reasonably expected in mindfulness intervention studies [58]. A sample size of 40 with a 25% attrition rate will give an estimated sample of 30 subjects completing the 8-week program.

The approximate number of IBD patients is 600 in NHS Highland and 1,741 at NHS Grampian. With estimated recruitment rates of IBD interventional studies ranging between 10% and 20% (234 to 468 between the two sides), a sample size of 40 is reasonably achievable.

### Analysis

Audio-recorded focus groups and interviews with practitioners will be transcribed verbatim. Transcripts will be analysed thematically using the framework analysis approach [59] which is a rigorous method that allows themes to be identified (and organised) within the groups or between the groups. Hence, notable differences in experiences and perceptions can be identified.

From quantitative data we will generate the following: estimates of eligibility, recruitment and retention rates, speed of recruitment, and completion rates of study assessment tools (objectives 1 and 4); Descriptive presentations of the proposed primary and secondary outcomes will be made to inform a sample size calculation for a large-scale trial and decisions as to whether their inclusion would be informative in a future trial (objective 3).

## Discussion

There is increasing evidence that mindfulness-based interventions can provide benefits to people with chronic ill health in terms of improving QoL and reducing anxiety and depressive symptoms. However, the use of these interventions in patients with both CD and UC is not researched. This paper outlines a protocol for a pilot RCT with embedded process evaluation that aims to provide data on eligibility, uptake and retention rates, barriers to recruitment and attendance and perceived benefit to IBD patients. This information is required to design a full-scale RCT assessing the effectiveness of MBCT on QoL for IBD patients.

This study is the first RCT to examine the use of MBCT in patients with IBD. The study design is a multicentred RCT and uses robust methods to evaluate feasibility and acceptability of the intervention in patients with IBD. The 6-month follow-up data will give an indication as to how long (if any) benefit from the intervention would last. The type of information that will be collected from the evaluation process will give an insight into important questions such as participants’ subjective thoughts and experiences about the intervention (expectations, potential benefits, barriers to attending and availability of the program through NHS) as well as their feedback on recruitment process and procedures.

In addition, the results of this project will provide important information on integration of mindfulness-based interventions with usual medical care as well as application of MBCT for IBD.

## Trial status

Recruitment commenced in May2013 and is ongoing.

## Abbreviations

BDI-II: Becks depression inventory; CD: Crohn’s disease; CDAI: Crohn’s disease activity index; IBD: Inflammatory bowel disease; IBDQoL: Inflammatory bowel disease quality of life; MAAS: Mindful attention awareness scale; MBCT: Mindfulness-based cognitive therapy; MBSR: Mindfulness-based stress reduction; MRC: Medical Research Council; QoL: Quality of life; RCT: Randomised controlled trial; SCCAI: Simple clinical colitis activity index; STAI: State and trait anxiety inventory; UC: Ulcerative colitis.

## Competing interests

The authors declared that they have no competing interests.

## Authors’ contributions

All authors have contributed to the design of the study and the preparation of the draft manuscript. MS as chief investigator and grant holder, co-conceived the study, drafted the study protocol and study materials, applied for ethics and NHS R&D approvals, and drafted the manuscript; AJMW co-conceived the study and participated in the design of the study and study materials, statistical planning and revision of the manuscript. GH contributed to the design of the study, particularly with the conceptualisation of the theory for the process evaluation and commented on the draft manuscript. IA provided the statistical analysis plan and commented on the draft manuscript. All authors read and approved the final manuscript.
